# Neighbourhood drivability: environmental and individual characteristics associated with car use across Europe

**DOI:** 10.1186/s12966-019-0906-2

**Published:** 2020-01-17

**Authors:** Nicolette R. den Braver, Julia G. Kok, Joreintje D. Mackenbach, Harry Rutter, Jean-Michel Oppert, Sofie Compernolle, Jos W. R. Twisk, Johannes Brug, Joline W. J. Beulens, Jeroen Lakerveld

**Affiliations:** 1 Department of Epidemiology and Biostatistics, Amsterdam University Medical Centers, Vrije Universiteit Amsterdam, Amsterdam Public Health research institute, Amsterdam, Amsterdam, The Netherlands; 2 Department of Social and Policy Sciences, University of Bath, Bath, UK; 3 Department of Nutrition Pitié-Salpêtrière, Sorbonne Université; Institute of Cardiometabolism and Nutrition, Hospital, Assistance Publique-Hôpitaux de Paris, Paris, France; 4 Equipe de Recherche en Epidémiologie Nutritionnelle (EREN), Université Paris 13, Centre de Recherche en Epidémiologie et Statistiques, Inserm (U1153), Inra (U1125), Cnam, COMUE Sorbonne Paris Cité, Bobigny, France; 5 Department of Movement and Sport Sciences, Faculty of Medicine and Health Sciences, Ghent University, Ghent, Belgium; 6 National Institute for Public Health and the Environment (RIVM), Bilthoven, The Netherlands; 7 The Netherlands and the Amsterdam School for Communication Research, University of Amsterdam, Amsterdam, The Netherlands; 8 Julius Center for Health Sciences and Primary Care, University Medical Center Utrecht, Utrecht, The Netherlands; 9 Global Geo Health Data Center, Utrecht University, Utrecht, The Netherlands

**Keywords:** Built environment, Passive transport, Active transport, Drivability, Sustainability, Transport, Urban health

## Abstract

**Background:**

Car driving is a form of passive transportation associated with higher sedentary behaviour, which is associated with morbidity. The decision to drive a car is likely to be influenced by the ‘drivability’ of the built environment, but there is lack of scientific evidence regarding the relative contribution of environmental characteristics of car driving in Europe, compared to individual characteristics. This study aimed to determine which neighbourhood- and individual-level characteristics were associated with car driving in adults of five urban areas across Europe. Second, the study aimed to determine the percentage of variance in car driving explained by individual- and neighbourhood-level characteristics.

**Methods:**

Neighbourhood environment characteristics potentially related to car use were identified from the literature. These characteristics were subsequently assessed using a Google Street View audit and available GIS databases, in 59 administrative residential neighbourhoods in five European urban areas. Car driving (min/week) and individual level characteristics were self-reported by study participants (analytic sample *n* = 4258). We used linear multilevel regression analyses to assess cross-sectional associations of individual and neighbourhood-level characteristics with weekly minutes of car driving, and assessed explained variance at each level and for the total model.

**Results:**

Higher residential density (β:-2.61, 95%CI: − 4.99; -0.22) and higher land-use mix (β:-3.73, 95%CI: − 5.61; -1.86) were significantly associated with fewer weekly minutes of car driving. At the individual level, higher age (β: 1.47, 95%CI: 0.60; 2.33), male sex (β: 43.2, 95%CI:24.7; 61.7), being employed (β:80.1, 95%CI: 53.6; 106.5) and ≥ 3 person household composition (β: 47.4, 95%CI: 20.6; 74.2) were associated with higher weekly minutes of car driving. Individual and neighbourhood characteristics contributed about equally to explained variance in minutes of weekly car driving, with 2 and 3% respectively, but total explained variance remained low.

**Conclusions:**

Residential density and land-use mix were neighbourhood characteristics consistently associated with minutes of weekly car driving, besides age, sex, employment and household composition. Although total explained variance was low, both individual- and neighbourhood-level characteristics were similarly important in their associations with car use in five European urban areas. This study suggests that more, higher quality, and longitudinal data are needed to increase our understanding of car use and its effects on determinants of health.

## Introduction

Car driving is a form of passive transportation associated with sedentary behaviour, which is in turn associated with morbidity [1]. Recent estimates indicate that 56% of the adult population across 28 European countries use a private car for daily trips whereas only 16% cycle or walk [1].

In previous studies car use has been associated with adverse health outcomes [2–5]. One study showed that substantial car use (> 10 h per week) was associated with a 50% higher risk of cardiovascular disease mortality [2]. Other studies have found associations between car driving and higher rates of obesity [3–5]. Driving to work was associated with 13% higher odds of obesity (95% CI 1.01; 1.27)) [3], and driving > 120 min per day was associated with 78% higher odds of obesity (95%CI: 1.61; 1.97) [4], in Australia. Additionally, a US modelling study indicated a 2.2% increase in obesity prevalence over 6 years, if each licensed driver increased their car travel by a mile per day [5]. Shifting from car use to active transport may therefore improve population health, and the built environment - an important determinant of travel behaviour - play a role in this shift to more active transport and health promotion. For example, a recent meta-analysis of observational studies indicated that highly walkable neighbourhoods – i.e. neighbourhoods featuring characteristics that promote walking – are associated with lower risk of type 2 diabetes [6]. Gaining insight into characteristics related to car driving can help inform transport-policies, and thereby improve health and outcomes such as traffic safety, air quality, congestion and climate change.

Walkability and green space, characteristics of the built environment, are consistently associated with higher levels of active transport in Europe [7–10]. However, individual and built environment characteristics specifically associated with car use and their relative contributions are less clear, especially across European countries. Car use may be influenced by a combination of individual and environmental characteristics. Previous studies have indicated that at the individual level, higher age, male sex, larger household composition, and being employed were associated with higher car use, and high socioeconomic status was consistently linked with both car ownership and car use [4, 11–15]. Built environmental characteristics were associated with car use include residential density, land-use mix, street network design, distance to destinations, parking availability and cost [13–24]. A meta-analysis showed that a 10% increase in road density, intersection density, access to jobs by car, distance to downtown and land-use mix, population density, access to job by transit or distance to transit were associated with 0.5–2.2% lower vehicle miles travelled [11]. Another study observed that a $6 increase in parking cost was associated with 16% lower probability of car use [24]. However, these studies were mainly non-European, and studies on potential determinants of car use in European settings are scarce.

Studies on potential determinants of car use in European settings, and especially across European countries, are scarce. Moreover, not many studies compared the contribution of individual characteristics to car use with neighbourhood characteristics. One study investigated the association between demographic and built environment variables with car ownership and daily travel by car, while drawing a comparison between the US and the UK. This study observed overall higher vehicle miles travelled by males, younger adults, employed individuals, and people with higher incomes. Correlates of car use were different for both settings, where socioeconomic status was more strongly associated with car use in the UK, the higher income groups travelled 5.6 vehicle miles more, compared to 2.4 vehicles miles in the highest income category in the US. In the US the highest population density category (10.000 persons/mile^2^) was associated with 8.0 fewer vehicle miles travelled per day, while in the UK the reduction was 1.6 vehicle miles [12].

Therefore, the aim of this study was to explore correlates of car driving in adults from five urban areas across Europe. We answered the following two research questions:

1. Which individual and neighbourhood characteristics are associated with car driving (in minutes per week), in five urban areas across Europe?
2. What percentage of variance in car driving minutes per week is explained by these individual- and neighbourhood-level characteristics?

## Methods

### Evidence-derived characteristics

Based on the available literature, a list of candidate variables important for ‘neighbourhood drivability’ was identified, and categorized according to the six D’s classification of Ewing & Cervero [11]. This classification originates from transport research and serves to identify influences in the built environment that potentially moderate travel demand. The classification consists of: density, diversity, design, destination accessibility, distance to transit and demand management [25]. The list of built environment variables was narrowed down to variables for which data sources could reasonably be obtained in a cross-European setting. The resulting selection of built environment characteristics include residential density, population density, car road density, land-use mix, traffic signal density, intersection density, parking at work, distance to destination, distance to transit, parking supply, parking cost, and are summarized and defined in Table 1.

**Table 1:** Environmental characteristics associated with time spent in passive transport modes based on the literature, with their implied relation to car driving

Environmental characteristics	Implied relation with drivability
Density	
Residential density^a^	Higher residential density associated with lower car use [14–16].
Population density	Higher population density associated with lower car ownership and car use [15–17].
Car road density^a^	Higher car road density associated with a higher car use [13].
Diversity	
Land-use mix^a^	Higher diversity associated with lower car use [14, 18, 26].
Design	
Traffic signal density^a^	Higher traffic signal density associated with lower car use [18].
Intersection density	Higher three-way intersection density associated with higher car use [19].
Destination accessibility	
Parking at work	Higher availability of parking at work associated with higher car use [27].
Distance to destination	Higher distance to work or retail, associated with higher car use [20, 28].
Distance to transit	
Distance to transit	Higher distance to transit associated with higher car use [17, 18, 29].
Demand management	
Parking supply^a^	Higher availability of parking lots is associated with higher car use [17, 21, 22, 30].
Parking costs	Higher parking cost is associated lower car use [23].

^a^Variables included in the present study

### Study design

For this study we used data from the Sustainable Prevention of Obesity Through Integrated Strategies (SPOTLIGHT) study. Details of this study are described elsewhere [31, 32]. In short, a neighbourhood audit and an individual-level survey were conducted in 60 randomly selected urban neighbourhoods from five European countries (Belgium, France, Hungary, the Netherlands and the United Kingdom). The urban areas were: Ghent and suburbs in Belgium; Paris and suburbs in France; Budapest and suburbs in Hungary; the Randstad (a conurbation including Amsterdam, Rotterdam, the Hague and Utrecht) in the Netherlands, and Greater London in the United Kingdom. In each of these urban areas, 12 neighbourhoods were selected, ensuring variety in residential area density (high and low density: > 2/3 and < 1/3 of area covered by residential buildings, respectively) and socioeconomic status (SES) (high and low: third and first tertile of neighbourhood level income, respectively) at neighbourhood level. Details on sampling can be found elsewhere [32]. A random sample of inhabitants (≥18 years) was invited to participate in the online survey, 6037 participants were included in the SPOTLIGHT study (response rate: 10.8%) [32].

For the present study, we excluded participants with missing (*n* = 530) and extreme values (*n* = 82) (z-score > 3) on the dependent variable car driving minutes per week and those with missing address or neighbourhood audit variables (*n* = 838). A complete-case analysis was performed due to the low proportion of missing values in covariates (9%), resulting in an analytical sample for the main analyses of *n* = 4258 (total sample descriptions are presented in Additional file 1: Table S1).

### Neighbourhood environmental characteristics

Environmental characteristics were measured at the neighbourhood level, defined by local administrative boundaries, except for Hungary. Budapest is officially divided into districts and suburbs that are much larger and contain a much more heterogeneous population than the administrative areas in the other study countries. Therefore, to ensure comparability between study areas, neighbourhoods in Budapest and suburbs were defined as 1 km^2^ areas [32]. On average a neighbourhood consisted a mean population of 2700 inhabitants in an area of 1,5km^2^. Neighbourhood definitions and characteristics are described in detail in a previously published paper [32].

Neighbourhood characteristics were assessed by the SPOTLIGHT virtual audit tool [33], a virtual street audit, using Google Street View to assess characteristics of physical activity- and food environment. In addition we used open data sources to derive additional characteristics that could be linked to cross-country respondent’s residential postal codes. Using these sources, we obtained a selection of the variables listed in Table 2: residential density, car road density, land-use mix, traffic signal density, and parking supply. The details of collection, calculations and use of these data are described below, according to an adapted version of the Geo-FERN reporting framework (Additional file 2: Table S2).

**Table 2:** Descriptive statistics of individual- and neighbourhood environmental characteristics of all respondents, and stratified by country

	Total	Belgium	France	Hungary	The Netherlands	UK
	*n* = 4258	*n* = 1382	*n* = 477	*n* = 641	*n* = 1311	*n* = 447
Individual characteristics						
Age	51.1 (15.9)	51.8 (16.2)	48.7 (14.)	48.2 (15.4)	53.8 (15.3)	48.4 (17.1)
Gender (female, %)	54.9	52.2	55.8	61.9	53.2	57.1
Employment (%)						
Currently employed	57.4	53.5	67.1	56.3	56.4	63.3
Currently not employed	15.7	15.9	14.2	17.9	15.9	13.4
Retired	26.9	30.6	18.7	25.7	27.8	23.3
Household composition (%)						
1-persons	21.4	17.2	21.2	18.7	24.1	30.2
2-persons	39.9	43.8	28.1	33.2	43.7	38.9
3-or-more-persons	38.7	39.0	50.7	48.1	32.2	30.9
Education (% higher)	55.9	47.9	74.2	51.0	57.5	63.3
Car driving (min/week)	265.8 (322.2)	370.4 (366.0)	214.4 (287.4)	164.6 (257.5)	270.2 (304.5)	129.4 (224.9)
Environmental characteristics						
Car road density (%)	11.6 (4.38)	11.2 (4.12)	12.3 (2.44)	9.7 (3.21)	13.4 (4.77)	9.63 (4.83)
Residential density (%)	61.1 (15.2)	59.8 (13.2)	58.5 (19.8)	65.4 (13.9)	64.7 (11.6)	49.8 (19.1)
Land-use mix (entropy-score^*a*^)	36.3 (19.0)	32.0 (12.0)	40.1 (27.8)	38.5 (14.6)	31.1 (17.5)	57.3 (19.3)
Traffic signal density (%)	20.1 (12.9)	18.4 (11.1)	41.8 (9.8)	5.9 (5.4)	22.1 (8.0)	16.3 (6.9)
Parking supply (n/km^2^)	11.4 (16.4)	6.3 (8.52)	15.3 (21.5)	11.9 (13.6)	15.6 (21.6)	9.93 (7.52)

Values between brackets are the Standard Deviations

^a^The entropy score ranges from 0 to 100 and is normalised using natural logarithm of the number of land uses (i.e. 1 Industrial, commercial, public, military and private units, 2 Residential areas, 3 Green urban areas, and 4 Sports and leisure facilities)

#### Density

Car road density was defined as the percentage of area coverage of fast transit and other roads and associated land per neighbourhood [11]. Residential density was defined as percentage of the area coverage of residential buildings per neighbourhood [11]. Data were obtained from the Urban Atlas (European Environment Agency, 2002), a Geographic Information System (GIS) database distributed by the European Environmental Agency, which provided high-resolution satellite image data on land use across Europe [34, 35]. The purpose of the European Environment Agency is to provide high quality data and independent data on the environment (e.g. greenhouse gas emissions, heavy metals in water, land-use). Car road and residential densities were obtained for the five urban areas under study, by intersecting land-use layers with neighbourhood boundaries, in ArcGIS version 10.6, resulting in a percentage of neighbourhood area devoted to car roads or residential area. Density variables ranged from 0 to 100%, with higher values indicating a higher density.

#### Diversity

Land-use mix was defined as heterogeneity in land uses in a given area [18]. Land use data were derived from the Urban Atlas, as described above, and four land-use categories were included, according to categories predetermined by the Urban Atlas: 1) Industrial, commercial, public, military and private units, 2) Residential areas, 3) Green urban areas, and 4) Sports and leisure facilities. Land-use mix was measured by means of an entropy index (Eq. 1). This entropy index is normalized using the natural logarithm of the number of land uses, and multiplied by 100 [36]. The entropy index was obtained per administrative neighbourhood and ranged from 0 to 100, with higher values indicating higher diversity.





#### Design

Traffic signal density was obtained by neighbourhood audit using the validated SPOTLIGHT-Virtual Audit Tool (S-VAT) [33]. The S-VAT enabled a standardised exposure assessment for cross-country comparison, and was based on existing tools [33]. For the current study, two parameters of traffic signal density were available: 1) Traffic calming devices, including speed humps, traffic islands, roundabouts and traffic lights, and 2) Pedestrian crossings, including zebra-paths and traffic lights. The criterion validity of these elements were very high (range: 89.9–96.9%), inter-observer reproducibility was good to excellent (range 68.8–95.3%), intra-observer reproducibility was excellent (89.8–96.9%) [33]. All streets in the residential neighbourhood were audited, as per availability of Google Street View data at the time of the study. The count of traffic calming devices and pedestrian crossings was obtained per street segment during the audit. The proportion of street segments with at least one traffic signal compared to the total number of street segments was calculated within each administrative neighbourhood. The traffic signal density ranged from 0 to 100, with higher values indicating higher traffic signal density.

#### Demand management

Parking data were obtained in May 2018 from OpenStreetMap (OSM), an open data source where non-commercialised users uploaded data in an online map. The purpose of OSM is to provide a free and editable map at global scale, with local knowledge and expertise. Data collection methods include field audits but also remote sensing, depending on data availability and the choices by the uploader, leading to heterogeneity in data quality. Notwithstanding these limitations, OSM provides data that are not available from traditional GIS sources on a global scale. All available parking facilities identified in OSM were off-street parking facilities. Two variable types were used for parking facilities across the included urban regions: polygons (parking surface in square meter) and point locations (x, y coordinates of parking facilities). To harmonize surfaces and locations across countries, polygons were transformed to centroid point locations, in ArcGIS version 10.6. The proportion of the total number of parking locations to the total surface area was calculated per administrative neighbourhood. Parking density was expressed as the number of parking locations per km^2^.

### Individual characteristics

Age, sex, employment status, household composition, and education were obtained from the SPOTLIGHT survey. Employment was categorized into currently employed, currently not employed or retired. Household composition was categorized into household with 1-person, 2-persons or 3-or-more persons. Education was self-reported in the survey with multiple but differing categories in each country [32]. We combined these categories to classify the education level of participants as either higher (college or university level) or lower (below college level).

### Car use

Self-reported car driving minutes per week were assessed in the online SPOTLIGHT survey. The survey collected information on mode of transportation in commuting and non-commuting trips, average duration of commute and non-commute per day and how many days per week these trips were taken. For this study, trip durations per day for commuting and non-commuting trips were summed. The total weekly car minutes were calculated by multiplying the questions ‘the number of days per week commute by car/moped in the last seven days’ and ‘the time spent (minutes/hours) on one of those days’. Car driving minutes per week were included in the analyses as a continuous variable. We performed sensitivity analyses to investigate differences in associations between individual and neighbourhood variables and car use, stratified by commuting and non-commuting travel (Additional file 3: Table S3).

### Statistical analysis

Socio-demographic and neighbourhood characteristics were summarized as proportions, means and standard deviations. Characteristics were presented for the total sample and by country.

To assess the associations between individual and neighbourhood environmental characteristics with car driving (min/week), linear mixed model analyses were performed, adjusted for clustering within neighbourhoods by adding a random intercept on neighbourhood level to the models. Non-standardised regression coefficients (β) and 95% Confidence intervals (95%CI) were reported as effect estimates. An intra-class correlation coefficient (ICC) was calculated according to the formula: variance_neighbourhood_ /(variance_individual_ + variance_neighbourhood_). For continuous variables deviations from linearity were checked, but none were detected.

To assess the relative contributions of individual- and neighbourhood level characteristics to the variance in car driving minutes per week, we first constructed an unconditional model without predictors to assess the total unexplained variance. Three conditional models were then constructed separately: Model 1 including individual-level variables, Model 2 including neighbourhood environmental-level variables, and Model 3 including both. Explained variance was calculated in these three models relative to the unconditional model, according to methods by Snijders & Bosker [38]. As neighbourhood-level determinants cannot explain variance in an individual level outcome, the variance component is split into individual-level car driving minutes per week (explained by individual level determinants) and neighbourhood level car driving minutes per week (explained by individual and neighbourhood level determinants). To compare the proportion of variance explained by individual characteristics, neighbourhood characteristics and both, we assessed the total model performance by looking at the reduction in unexplained variance for the total model. The total unexplained variance was a sum of the unexplained variance components on individual and neighbourhood level, divided by the total unexplained variance in the unconditional model. This resulted in a percentage variance reduction to compare the model performance when adding individual and neighbourhood characteristics. Second, we compared individual and neighbourhood characteristics in explaining the variation in neighbourhood level car driving. As a sensitivity analyses, the models were stratified by country to identify country specific patterns. Analyses were performed in STATA version 14.

## Results

Descriptive statistics are summarized in Table 2. Participants were on average 51.1 ± 15.9 years old, slightly more often female (54.9%) than male and employed (57.4%) than unemployed or retired. The total sample (*n* = 6.037) was similar to the study population in age, gender distribution, and household composition, but relatively fewer were currently employed, and fewer highly educated. Participants spend approximately 266 (±322) minutes per week in car driving. The ICC was 0.12, indicating clustering of car driving time within neighbourhoods. Descriptive statistics of neighbourhood characteristics per neighbourhood are included in Additional file 4: Table S4.

### Individual and neighbourhood characteristic associated with car driving

Each additional year of age (β: 1.47, 95%CI: 0.60; 2.33), male sex (β: 42.4, 95%CI: 24.7; 61.7), employed, compared to unemployed, (β:80.1, 95%CI: 53.6; 106.5) and living in households of ≥3 persons, compared to a one-person household (β: 47.4, 95%CI: 20.6; 74.2) were associated with more minutes of driving per week. Education was not significantly associated with minutes of driving per week (Table 3).

**Table 3:** Associations between individual- and neighbourhood environmental characteristics with car driving (min/week) (*n* = 4258)

	Model 1		Model 2		Model 3	
	*B* (SE)	*p*	*B* (SE)	*p*	*B* (SE)	*p*
Individual characteristics						
Age	1.47 (0.44)	0.001			1.43 (0.44)	0.001
Gender (man)	43.2 (9.44)	< 0.001			43.1 (9.44)	< 0.001
Employment (employed)	80.1 (13.5)	0.000			80.0 (13.5)	< 0.001
Household composition (≥3)	47.4 (13.7)	0.001			45.9 (13.7)	0.001
Education (high)	−14.2 (10.2)	0.164			−14.5 (10.2)	0.156
Neighbourhood characteristics						
Car road density^a^			−5.16 (3.53)	0.144	−3.85 (3.42)	0.260
Residential density^b^			−2.62 (1.25)	0.036	−2.47 (1.21)	0.041
Land-use mix^c^			−3.82 (0.98)	0.000	−3.56 (0.95)	< 0.001
Traffic signal density^d^			0.81 (1.05)	0.436	0.68 (1.01)	0.504
Parking supply^e^			−0.12 (0.88)	0.890	−0.26 (0.86)	0.760
Explained variance at individual level	**0.03**		**–**		**–**	
Explained variance at neighbourhood level	**0.09**		**0.25**		**0.30**	
Total unexplained model variance	**0.98**		**0.97**		**0.95**	

Model 1: Includes individual characteristics age, gender, employment, household composition and education

Model 2: Includes neighbourhood characteristics car road density, residential density, land-use mix, traffic signal density and parking supply

Model 3: Includes individual characteristics age, gender, employment, household composition and education, and neighbourhood characteristics car road density, residential density, land-use mix, traffic signal density and parking supply

^a^ Percentage of coverage of fast transit roads and associated land, and other roads and associated land per neighbourhood

^b^ Percentage of coverage of buildings devoted to residential facilities per neighbourhood

^c^ Entropy-score of 1) Industrial, commercial, public, military and private units, 2) Residential areas, 3) Green urban areas, and 4) Sports and leisure facilities

^d^ Traffic signal density (including traffic calming devices and pedestrian crossing) per street segment per neighbourhood

^e^ Percentage of parking lots per neighbourhood

^f^ Explained variance was obtained relative to the unconditional model, for model 1 residual variance (level1) and intercept variance (level 2) are presented, and for model 2 only intercept variance is presented

Higher residential density (β: -2.61, 95%CI: − 4.99; −0.22) and higher land use mix (β: -3.73, 95%CI: − 5.61; −1.86) were significantly associated with fewer minutes of driving per week. Road density, parking supply and traffic signal density were not significantly associated with minutes of driving per week (Table 3).

For non-commute trips, the same associations were observed as in all trips, although residential density became just non-significant. For commute trips, we observed that mainly males and those who were employed were likely to drive, while age and household composition were not significantly associated anymore. On the neighbourhood level, the similar associations were observed (Additional file 3: Table S3).

### Variance explained by individual and neighbourhood characteristics

All variables in the model reduced the total model unexplained variance by 5%, where individual-level characteristics accounted for 2% and neighbourhood-level characteristics for 3%. Variation in neighbourhood-level car driving was explained for 9% by individual characteristics, whereas 30% was explained by adding neighbourhood characteristics (Table 3). This is an indication that variation in car use across neighbourhoods is for a large part determined by neighbourhood characteristics, rather than individual characteristics.

### Sensitivity analyses – per country

Neighbourhood clustering in minutes of driving per week was highest in France (ICC = 0.15), and lowest in Belgium and Hungary (ICC = 0.03). The total model unexplained variance reduction was highest in the UK (18%), and lowest in The Netherlands (4%). In the main analyses we observed that this reduction was about twice as large when neighbourhood variables were included. A sensitivity analyses indicated that this was especially the case in Belgium, Hungary and The Netherlands, while adding neighbourhood characteristics made less of a difference in France and the UK (France: 6 to 8%, UK: 15 to 18%).

Neighbourhood level car driving minutes, the explained variance by individual variables ranged from 9% (Hungary) to 44% in the UK, and ranged from 26% in France to 74% in Belgium by the combination of both individual and neighbourhood characteristics (Additional file 5: Table S5).

## Discussion

We studied the association of a range of individual and neighbourhood characteristics with reported car driving time across five urban regions in Europe. We investigated which individual- and neighbourhood level characteristics were associated with car driving minutes per week and explored what percentage of variance in car driving minutes per week was explained by individual- and neighbourhood-level characteristics. First, we found that younger age, female sex, being unemployed, and living in a smaller household were associated with less car driving minutes per week, and at the neighbourhood level higher residential density and land-use mix were associated with less car minutes per week. The total model explained 5% of the model variance when neighbourhood and individual characteristics were combined, and these contributed almost equally. Variation in neighbourhood level car use was explained for 9% by individual characteristics, and 30% by both individual and neighbourhood characteristics, indication that variation in car use across neighbourhoods is for a large part determined by neighbourhood characteristics. Previous research on relations between the built environment and car use has mainly been performed in non-European settings. This study confirms key environmental characteristics across Europe, and provides insights into the importance of studying the ways in which the built environment influences behaviour. To our knowledge, our study was the first attempt to assess the importance of neighbourhood characteristics in comparison to individual characteristics in explaining car driving.

Our findings are in line with previous literature reporting that older age, male sex, larger household composition, and being employed are associated with higher car use [4, 11–15]. However, high socioeconomic status was most consistently linked with both car ownership and car use [4, 11–15], while in our study only unemployment was associated with lower car use, but not education. One explanation could be that we lost sensitivity in our education variable, because it was a dichotomous variable. Regarding built environmental characteristics, our study found that higher residential density and land-use mix were statistically significantly associated with lower car use, which is in line with previous research. Compared to elasticities in car use from a meta-analysis including mainly North-American studies (0.9 and 2.2% respectively) [11], this study indicated that a 10% increase in residential density and land use mix were associated with 5.7 and 4.9% lower car use in this cross-European setting. Road density was non-significantly associated with 1.6% lower car use compared to 1.2% in literature [11]. In addition, the findings correspond to studies that observed a positive association between neighbourhood walkability and higher levels of walking or active transport [7–10]. Walkability indices usually include variables that capture residential density, land-use mix and connectivity, and this study confirms the inverse association for the first two indicators with car use.

The variance explained by the total model (5%) was in line with previous studies. For example, the walkability index explained 8,3% of variation in active transport, whereas individual’s income explained 1.1% [39, 40]. Another study performed in the US and UK found 16% of explained variance in total daily travel distance by individual characteristics, resources for transportation, and neighbourhood characteristics together [12]. No distinction was made between these three variance sources, but the associations for income were stronger for individuals in UK (UK daily vehicle miles β: 5.6, *p* < 0.05 vs US daily vehicle miles β: 2.4, p < 0.05) and stronger for residential density in US (US daily vehicle miles β: − 8.0, *p* < 0.05 vs UK daily vehicle miles β: 1.6, p < 0.05). None of these studies made a comparison in variance explained by individual level variables compared to neighbourhood level variables.

The total explained variance of our model was relatively low, which can be explained by two main arguments. First, we included information on residential neighbourhood characteristics, and were not able to include information on the destination characteristics or distance to work in our study, while this may reflect an important incentive of car use [20, 27, 28]. However, despite the additional relevance to study destination environments (such as the working environment), the home environment often is a start and/or end-point, and therefore of importance in transport mode choice. Moreover, the environmental characteristics within the neighbourhood may influence whether individuals use the car for short trips within their neighbourhood. If the neighbourhood environment is supportive to car use, this may enhance car trips for short distances, which could otherwise easily be replaced by active transport forms. Second, exposure misclassification may have led to lower explained variance. In the administrative neighbourhoods that were used for the exposure area, participants could have lived in the middle of their neighbourhood or on the edge [41]. This may have led to exposure misclassification in some individuals. However, because this is likely to be random misclassification across neighbourhoods, associations might have been attenuated, such that in reality associations could be stronger. Also, we may have found a higher variance explained if cost of car use was included. One study in the US included price variables, land use and individual characteristics which resulted in 69% explained variance in transport mode choice [24].

Country specific analyses showed a substantial heterogeneity in explained variance across the five urban areas. The neighbourhood explained variance within countries was much higher than in the overall analyses, probably because the variation between neighbourhoods within the same countries is lower than between countries. Therefore, the percentage of explained variance by neighbourhood characteristics is automatically higher within countries than in the overall analyses. The neighbourhood-level variance component should thus be interpreted to compare between countries, rather than comparing to the overall analyses across countries. In France this variance was low relative to the other countries, which may be an indication of neighbourhood variation being larger in France, and/or of data quality issues, such as the inconsistent OSM data inputs. OSM data is generated by non-commercialised users with varying level of experience and data was potentially entered with varying precision across countries [42]. In addition, parking supply can be defined as on-street parking, off-street parking, or home parking (e.g. households with their own garage or driveway) [43]. Due to limited data availability on private parking spaces, we included only off-street parking, while this may not be a valid reflection of the actual parking supply used at home. Studies demonstrated that the absence of a dedicated parking space at home, and longer walking distance to a parking facility, reduced the probability of car use [17, 44]. On the other hand, households with home parking generally own more cars, tend to make more car trips and are more likely to commute by car [45, 46].

Limitations of this study should be noted. Several potentially relevant environmental characteristics were not available in a harmonised way for all countries under study, such as distance to transit, distance to work, cost of car use, parking cost and parking pressure. Also, destination and route characteristics may be important for car use, which we could not include in our study. As discussed earlier, these factors may have led to a lower explained variance in car driving. Secondly, a potential bias that we could not address is self-selection bias. A recent study suggested that self-selection factors may affect associations between walkability and physical activity (in the residential neighbourhood, but also non-residential areas) [47], and it is likely that this may also apply for drivability. Finally, a study limitation was that our outcome, car minutes per week, was self-reported and the questionnaire item was not validated. However, this measure was available for the large sample and measured in the same way across five countries.

A strength of this study was that it mostly used reliable, high-resolution Europe-wide land use data with uniform standards for all cities, which allowed us to compare land use patterns in different European urban areas [34]. Additionally, generalizability of the results was increased by the assessment of many neighbourhoods, with high- and low density, with high- and low socio-economic status across Europe [32].

Cross validation of our findings in different datasets and on different populations is recommended. Future studies should also consider investigating the addition of other environmental measures such as walkability, and exploring the relation with other outcomes such as passive/active transport ratio, sedentary behaviour, non-communicable diseases, air quality, traffic injuries, and traffic congestions. In addition, studies could focus on a broader conceptualization of drivability by including more or other potential characteristics that may influence drivability, such as distance to transit, distance to work, or assess the drivability at both the home, commuting and the work environment [18], parking pressure [43, 44] and safety.

## Conclusion

Younger adults, those unemployed, women and those in smaller households drove less. On the neighbourhood level, higher residential density and land-use-mix were associated with less car driving. Although a large proportion of the model variance remained unexplained, individual and neighbourhood characteristics were similarly important for driving in five European urban areas. This study demonstrates that reducing car use might require a built environment that reduces car dependency by ensuring that relevant destinations are within a reasonable range for people using active transport.

## Supplementary information

**Additional file 1: Table S1.** Descriptive statistics of total SPOTLIGHT population (*n* = 6037)

**Additional file 2: Table S2.** Geo-FERN framework

**Additional file 3: Table S3.** Associations between individual- and neighbourhood environmental characteristics with car driving for commuting and non-commuting (min/week)

**Additional file 4: Table S4.** Environmental characteristics per neighbourhood

**Additional file 5: Table S5.** Associations between individual and environmental characteristics with car driving (min/week), stratified by country

**Additional file 6.**

## Data Availability

The data is not deposited in publicly available repositories due to the rules of the SPOTLIGHT consortium. The data -or parts of the data- are available to be used by others, but under conditions as specified within the SPOTLIGHT data access committee. For more information, please contact Jeroen Lakerveld (j.lakerveld@amsterdamumc.nl).

## Abbreviations

CVD: Cardiovascular diseases
GIS: Geographic information systems
ICC: Intraclass correlation coefficient
OSM: Open street map
SES: Socio-economic status
SPOTLIGHT: Sustainable prevention of obesity through integrated strategies
S-VAT: SPOTLIGHT virtual audit tool
