# Supplementary material for: Neighbourhood drivability: environmental and individual characteristics associated with car use across Europe
Source: Int J Behav Nutr Phys Act. 2020 Jan 17;17:8. doi: 10.1186/s12966-019-0906-2 (PMC6967086; doi:10.1186/s12966-019-0906-2)
Supplement: Supplementary file 1 — Additional file 1: Table S1. Descriptive statistics of total SPOTLIGHT population (n = 6037) [file 12966_2019_906_MOESM1_ESM.docx]

Supplementary Table 4: Descriptive statistics of total SPOTLIGHT population (n=6037)

|  | **Analytic sample**  n=4,258 | **Missing in total sample** | **Total sample**  n=6037 |  |
| --- | --- | --- | --- | --- |
| *Individual characteristics* |  |  |  |  |
| **Age** | 51.1 ± 15.9 | 62 | 51.8 ± 16.4 |  |
| **Gender** (female, %) | 54.9 | 60 | 55.4 |  |
| **Employment** (%)  Currently employed  Currently not employed  Retired | 57.4  15.7  26.9 | 23 | 54.5  16.1  29.0 |  |
| **Household composition** (%)  1-persons  2-persons  3-or-more-persons | 21.4  39.9  38.7 | 606 | 20.4  39.3  38.1 |  |
| **Education** (% higher) | 55.9 | 597 | 48.2 |  |
| *Values between brackets are the Standard Deviations.*  *^a^Entropy score ranges from 0-1 and is normalised using natural logarithm of the number of land uses (i.e. 1* *Industrial,*  *commercial, public, military and private units, 2 Residential areas, 3 Green urban areas, and 4 Sports and leisure facilities).* | | | | |
