# Supplementary material for: Neighbourhood drivability: environmental and individual characteristics associated with car use across Europe
Source: Int J Behav Nutr Phys Act. 2020 Jan 17;17:8. doi: 10.1186/s12966-019-0906-2 (PMC6967086; doi:10.1186/s12966-019-0906-2)
Supplement: Supplementary file 3 — Additional file 3: Table S3. Associations between individual- and neighbourhood environmental characteristics with car driving for commuting and non-commuting (min/week) [file 12966_2019_906_MOESM3_ESM.docx]

Supplementary Table 3. Associations between individual- and neighbourhood environmental characteristics with car driving for commuting and non-commuting (min/week)

|  | Non-commute (n=4181)  Model 3 | | Commute (n=2564)  Model 3 | |  |
| --- | --- | --- | --- | --- | --- |
|  | ***B* (SE)** | ***p*** | ***B* (SE)** | ***p*** | |
| Individual characteristics |  |  |  |  | |
| Age | **1.74 (0.35)** | **<0.001** | 0.41 (0.41) | 0.31 | |
| Gender (woman) | **-17.8 (7.55)** | **0.02** | **-41.9 (8.52)** | **<0.001** | |
| Employment (unemployed) | **44.2 (10.7)** | **<0.001** | **-67.5 (19.1)** | **<0.001** | |
| Household composition (≥3) | **51.6 (10.9)** | **<0.001** | -4.1 (12.2) | 0.74 | |
| Education (high) | -15.1 (8.1) | 0.06 | -3.54 (9.45) | 0.71 | |
| Neighbourhood characteristics |  |  |  |  | |
| Car road density^a^ | -1.23 (2.13) | 0.56 | -3.69 (2.38) | 0.12 | |
| Residential density^b^ | -1.44 (0.75) | 0.06 | **-1.72 (0.85)** | **0.04** | |
| Land-use mix^c^ | **-1.99 (0.60)** | **0.001** | **-2.41 (0.66)** | **<0.001** | |
| Traffic signal density^d^ | 0.25 (0.64) | 0.70 | 0.85 (0.71) | 0.24 | |
| Parking supply^e^ | -0.32 (0.54) | 0.55 | 0.05 (0.62) | 0.94 | |
