# Supplementary material for: Neighbourhood drivability: environmental and individual characteristics associated with car use across Europe
Source: Int J Behav Nutr Phys Act. 2020 Jan 17;17:8. doi: 10.1186/s12966-019-0906-2 (PMC6967086; doi:10.1186/s12966-019-0906-2)
Supplement: Supplementary file 4 — Additional file 4: Table S4. Environmental characteristics per neighbourhood [file 12966_2019_906_MOESM4_ESM.docx]

Supplementary Table 1. Environmental characteristics per neighbourhood.

| **Neighbourhood number**  **(n = 59)** | **Car road density (%)^1^** | **Residential density (%)^2^** | **Land-use mix (entropy score)^3^** | **Traffic signal density (%)^4^** | **Parking supply (n/km^2^)^5^** |
| --- | --- | --- | --- | --- | --- |
| **Belgium** |  |  |  |  |  |
| **1** | 20,97 | 66,66 | 0,23 | 0,56 | 1,66 |
| **2** | 13,38 | 81,95 | 0,18 | 0,39 | 11,14 |
| **3** | 9,21 | 50,68 | 0,32 | 0,51 | 5,36 |
| **4** | 10,57 | 63,43 | 0,41 | 0,30 | 1,04 |
| **5** | 5,61 | 53,13 | 0,19 | 0,06 | 0,00 |
| **6** | 8,22 | 43,50 | 0,31 | 0,11 | 0,00 |
| **7** | 10,72 | 81,34 | 0,22 | 0,25 | 4,79 |
| **8** | 4,14 | 55,42 | 0,28 | 0,97 | 0,00 |
| **9** | 10,04 | 69,82 | 0,29 | 0,84 | 0,00 |
| **10** | 16,75 | 69,80 | 0,31 | 0,98 | 8,27 |
| **11** | 11,84 | 46,80 | 0,29 | 0,48 | 0,00 |
| **12** | 12,69 | 44,98 | 0,57 | 0,98 | 25,30 |
| **The Netherlands** | |  |  |  |  |
| **13** | 14,90 | 64,80 | 0,40 | 0,51 | 78,82 |
| **14** | 7,08 | 78,00 | 0,11 | 0,36 | 0,63 |
| **15** | 13,25 | 74,28 | 0,33 | 0,59 | 5,87 |
| **16** | 11,70 | 75,85 | 0,11 | 1,24 | 15,39 |
| **17** | 20,59 | 75,39 | 0,00 | 0,87 | 14,96 |
| **18** | 7,85 | 61,44 | 0,29 | 0,77 | 2,35 |
| **19** | 12,40 | 67,28 | 0,36 | 0,40 | 14,12 |
| **20** | 20,63 | 65,22 | 0,39 | 1,12 | 12,52 |
| **21** | 15,81 | 44,17 | 0,72 | 0,80 | 2,67 |
| **22** | 21,34 | 60,98 | 0,26 | 0,61 | 0,00 |
| **23** | 5,10 | 35,82 | 0,45 | 0,63 | 11,85 |
| **24** | 12,10 | 55,85 | 0,38 | 0,69 | 17,74 |
| **United Kingdom** |  |  |  |  |  |
| **25** | 16,65 | 65,40 | 0,46 | 0,76 | 12,90 |
| **26** | 6,82 | 34,12 | 0,82 | 0,40 | 15,94 |
| **27** | 13,69 | 38,43 | 0,69 | 0,52 | 29,37 |
| **28** | 14,85 | 73,91 | 0,31 | 0,55 | 11,91 |
| **29** | 16,41 | 38,53 | 0,57 | 0,92 | 11,95 |
| **30** | 11,19 | 40,33 | 0,91 | 0,58 | 14,85 |
| **31** | 6,66 | 73,17 | 0,25 | 0,47 | 0,95 |
| **32** | 3,89 | 20,08 | 0,73 | 0,16 | 1,86 |
| **33** | 6,54 | 30,55 | 0,57 | 0,28 | 9,13 |
| **34** | 4,24 | 16,93 | 0,85 | 0,12 | 0,34 |
| **35** | 6,69 | 44,72 | 0,58 | 0,25 | 3,17 |
| **36** | 4,00 | 68,43 | 0,55 | 0,50 | 4,37 |
| **Hungary** |  |  |  |  |  |
| **37** | 15,57 | 63,98 | 0,42 | 0,59 | 28,00 |
| **38** | 14,41 | 62,86 | 0,61 | 0,39 | 7,00 |
| **39** | 4,52 | 40,24 | 0,52 | 0,07 | 4,50 |
| **40** | 6,22 | 89,42 | 0,07 | 0,02 | 1,63 |
| **41** | 11,92 | 73,32 | 0,31 | 0,03 | 9,00 |
| **42** | 6,11 | 61,79 | 0,44 | 0,17 | 1,00 |
| **43** | 9,04 | 40,81 | 0,66 | 0,32 | 43,00 |
| **44** | 12,07 | 77,67 | 0,26 | 0,18 | 1,00 |
| **45** | 10,22 | 76,00 | 0,35 | 0,17 | 20,00 |
| **46** | 6,72 | 59,77 | 0,33 | 0,02 | 1,67 |
| **47** | (excluded) |  |  |  |  |
| **48** | 7,72 | 72,25 | 0,29 | 0,08 | 1,00 |
| **France** |  |  |  |  |  |
| **49** | 16,35 | 26,95 | 0,79 | 1,32 | 97,33 |
| **50** | 11,94 | 49,01 | 0,60 | 1,46 | 18,38 |
| **51** | 15,36 | 72,10 | 0,38 | 1,29 | 0,00 |
| **52** | 11,81 | 57,03 | 0,40 | 1,62 | 0,00 |
| **53** | 9,53 | 86,43 | 0,13 | 0,70 | 0,00 |
| **54** | 15,16 | 82,95 | 0,08 | 1,80 | 41,13 |
| **55** | 13,38 | 64,92 | 0,49 | 1,03 | 7,32 |
| **56** | 8,74 | 59,76 | 0,64 | 1,51 | 36,91 |
| **57** | 8,53 | 47,64 | 0,00 | 1,00 | 12,78 |
| **58** | 12,39 | 30,58 | 0,76 | 1,56 | 27,04 |
| **59** | 13,12 | 86,88 | 0,00 | 1,21 | 0,00 |
| **60** | 16,01 | 75,18 | 0,24 | 1,15 | 0,00 |

*^1^ Percentage of coverage of fast transit roads and associated land, and other roads and associated land per neighbourhood.*

*^2^ Percentage of coverage of buildings devoted to residential facilities per neighbourhood.*

*^3^ Entropy-score of 1) Industrial, commercial, public, military and private units, 2) Residential areas, 3) Green urban areas, and 4) Sports and leisure facilities.*

*^4^ Traffic signal density (including traffic calming devices and pedestrian crossing) per street segment per neighbourhood.*

*^5^ Parking supply as number of parking locations per kilometre squared*

*^6^ Neighborhood level income in euro’s per year.*
