# Supplementary material for: Neighbourhood drivability: environmental and individual characteristics associated with car use across Europe
Source: Int J Behav Nutr Phys Act. 2020 Jan 17;17:8. doi: 10.1186/s12966-019-0906-2 (PMC6967086; doi:10.1186/s12966-019-0906-2)
Supplement: Supplementary file 5 — Additional file 5: Table S5. Associations between individual and environmental characteristics with car driving (min/week), stratified by country [file 12966_2019_906_MOESM5_ESM.docx]

| **Supplementary Table 2:** associations between individual and environmental characteristics with car driving (min/week), stratified by country. | | | | | | | | | | | | | | | | | | | | | | | | | | |
| --- | --- | --- | --- | --- | --- | --- | --- | --- | --- | --- | --- | --- | --- | --- | --- | --- | --- | --- | --- | --- | --- | --- | --- | --- | --- | --- |
|  |  | | **Belgium**  n=1,382, n_NBH_=12 | | | |  | **France**  n=477, n_NBH_=12 | | | |  | **Hungary**  n=641, n_NBH_=11 | | | |  | **The Netherlands**  n=1,311, n_NBH_=12 | | | |  | **UK**  n=447, n_NBH_=12 | | | |
| ICC | 0.03 | | |  |  | 0.15 | | |  |  | 0.03 | | |  |  | 0.04 | | |  |  | 0.07 | | |  |  |  |
|  | *B (SE)* | | | *p* |  | *B (SE)* | | | *p* |  | *B (SE)* | | | *p* |  | *B (SE)* | | | *p* |  | *B (SE)* | | | *p* |  |  |
| ***Model 1^a^*** |  | | |  |  |  | | |  |  |  | | |  |  |  | | |  |  |  | | |  |  |  |
| **Individual characteristics** |  | | |  |  |  | | |  |  |  | | |  |  |  | | |  |  |  | | |  |  |  |
| Age | 0.61 (0.91) | | | 0.502 |  | **3.36 (1.24)** | | | **0.007** |  | **3.29 (0.90)** | | | **0.000** |  | 0.22 (0.80) | | | 0.782 |  | **3.74 (0.91)** | | | **0.000** |  |  |
| Gender (woman) | -25.5 (19.4) | | | 0.190 |  | **-69.7 (24.4)** | | | **0.004** |  | **-76.1 (20.3)** | | | **0.000** |  | **-46.9 (17.0)** | | | **0.006** |  | 0.31 (20.5) | | | 0.988 |  |  |
| Employment (unemployed) | **-134.2 (28.5)** | | | **0.000** |  | -37.0 (35.6) | | | 0.299 |  | **-74.2 (27.2)** | | | **0.006** |  | **-73.0 (23.8)** | | | **0.002** |  | 36.7 (31.0) | | | 0.237 |  |  |
| Household composition (≥3) | 37.6 (30.1) | | | 0.212 |  | 49.9 (32.9) | | | 0.129 |  | **61.0 (28.6)** | | | **0.033** |  | 45.2 (24.2) | | | 0.061 |  | **90.3 (28.2)** | | | **0.001** |  |  |
| Education (higher) | -3.84 (20.7) | | | 0.853 |  | -10.4 (29.3) | | | 0.723 |  | 15.6 (20.3) | | | 0.442 |  | **-43.5 (18.2)** | | | **0.017** |  | -27.1 (22.6) | | | 0.231 |  |  |
| **Explained variance individual**  **Explained variance NBH** | **0.03**  **0.17** | | |  |  | **0.07**  **0.16** | | |  |  | **0.07**  **0.09** | | |  |  | **0.02**  **0.14** | | |  |  | **0.08**  **0.44** | | |  |  |  |
| **Reduction in total unexplained variance** | **0.02** | | |  |  | **0.06** | | |  |  | **0.06** | | |  |  | **0.02** | | |  |  | **0.15** | | |  |  |  |
| ***Model 2^c^*** |  | | |  |  |  | | |  |  |  | | |  |  |  | | |  |  |  | | |  |  |  |
| **Individual characteristics** |  | | |  |  |  | | |  |  |  | | |  |  |  | | |  |  |  | | |  |  |  |
| Age | -0.57 (0.91) | | | 0.531 |  | **3.39 (1.24)** | | | **0.006** |  | **3.32 (0.90)** | | | **0.000** |  | -0.09 (0.81) | | | 0.912 |  | **3.35 (0.96)** | | | **0.001** |  |  |
| Gender (woman) | -23.4 (19.4) | | | 0.228 |  | **-69.5 (24.4)** | | | **0.004** |  | **-74.4 (20.2)** | | | **0.000** |  | **-48.2 (17.0)** | | | **0.005** |  | 1.39 (20.5) | | | 0.946 |  |  |
| Employment (unemployed) | **-134.3 (28.5)** | | | **0.000** |  | -35.0 (35.6) | | | 0.326 |  | **-70.2 (27.2)** | | | **0.010** |  | **-76.0 (23.8)** | | | **0.001** |  | 38.3 (30.8) | | | 0.215 |  |  |
| Household composition (≥3) | 39.9 (30.1) | | | 0.185 |  | 49.0 (32.9) | | | 0.136 |  | **59.4 (28.9)** | | | **0.040** |  | 39.9 (24.4) | | | 0.102 |  | **71.1 (28.9)** | | | **0.014** |  |  |
| Education (higher) | -8.07 (20.8) | | | 0.698 |  | -10.9 (29.5) | | | 0.711 |  | 14.2 (20.0) | | | 0.478 |  | **-49.5 (18.2)** | | | **0.006** |  | -13.9 (23.0) | | | 0.544 |  |  |
| **Environmental characteristics** |  | | |  |  |  | | |  |  |  | | |  |  |  | | |  |  |  | | |  |  |  |
| Car road density | -5.04 (3.21) | | | 0.117 |  | -13.4 (13.1) | | | 0.308 |  | 4.38 (20.0) | | | 0.478 |  | **-5.89 (2.86)** | | | **0.040** |  | **-14.4 (5.04)** | | | **0.004** |  |  |
| Residential density | 0.52 (1.26) | | | 0.679 |  | -0.59 (2.64) | | | 0.822 |  | -2.28 (2.07) | | | 0.270 |  | -2.49 (1.72) | | | 0.148 |  | 0.37 (1.09) | | | 0.737 |  |  |
| Land-use mix | 2.81 (2.01) | | | 0.162 |  | -1.24 (1.79) | | | 0.489 |  | -0.31 (1.90) | | | 0.870 |  | **-2.90 (1.16)** | | | **0.013** |  | -0.24 (1.02) | | | 0.817 |  |  |
| Traffic signal density | -0.30 (1.32) | | | 0.822 |  | 1.76 (3.66) | | | 0.630 |  | -1.68 (3.48) | | | 0.629 |  | -1.34 (1.69) | | | 0.427 |  | 6.07 (3.2) | | | 0.056 |  |  |
| Parking supply | **-7.84 (2.33)** | | | **0.001** |  | 0.78 (1.61) | | | 0.629 |  | **-3.47 (1.10)** | | | **0.002** |  | 0.32 (0.63) | | | 0.615 |  | -0.80 (2.17) | | | 0.713 |  |  |
| **Explained variance individual**  **Explained variance NBH** | | **-**  **0.74** | |  |  | **-**  **0.26** | | |  |  | **-**  **0.62** | | |  |  | **-**  **0.60** | | |  |  | **-**  **0.70** | | |  |  |  |
| **Total reduction in unexplained variance** | **0.05** | | |  |  | **0.08** | | |  |  | **0.10** | | |  |  | **0.04** | | |  |  | **0.18** | | |  |  |  |
| ^a^ Model 1: Includes individual characteristics age, gender, employment, household composition and education.^b^ Model 2: Includes neighbourhood characteristics car road density, residential density, land-use mix, traffic signal density and parking supply.^c^ Model 3: Includes individual characteristics age, gender, employment, household composition and education, and neighbourhood characteristics car road density, residential density, land-use mix, traffic signal density and parking supply.^d^ Explained variance was obtained relative to the unconditional model for residual variance (level1) and intercept variance (level 2), according to Snijders & Bosker ^44^. | | | | | | | | | | | | | | | | | | | | | | | | | |  |
